# British Journal of Surgery

**Published:** 1913-09

**Authors:** 


### Reviews of Books.

**British Journal of Surgery.** Vol. I. No. 1. July, 1913. Bristol: John Wright & Sons Ltd. 7s. 6d. net.

We heartily welcome the appearance of the first number of the *British Journal of Surgery*. We have, of course, the *Annals of Surgery* in our own language, but it is essentially an American journal, and though the names of some of the writers are well known to English surgeons, yet many are not, and it greatly adds to the value of any communication to the reader if he knows something of the qualifications of the writer to form the opinions he expresses. Some of the largest continental countries have had Journals of Surgery for some years, and it was felt that this country should not be behind in this respect.

We think the editors and the publishers have produced a journal which will do great credit to British surgery. The articles in the first number are of much value, and deal with many advances in the surgical art. It is profusely and very well illustrated, and so printed as to be very easy to read.

The opening paper is by Dr. David Newman, of Glasgow, and is a very valuable contribution to the study of symptomless renal hæmaturia. The new methods of producing anæsthesia are fully considered by Dr. Shipway, an anæsthetist to Guy's Hospital, in an able paper ; and to the new method of intratracheal anæsthesia Mr. Kelly, of Liverpool, has devoted a very instructive article. Such topics are, of course, of vital importance to the surgeon. Amongst many other very interesting papers is one on " Rupture of the Crucial Ligaments and Fracture of the Spine of the Tibia," of which Mr. Robert Jones, of Liverpool, is joint contributor. It is exceedingly well illustrated by skiagrams. Perhaps many readers will turn at once to what must be one of the most interesting parts of the Journal—the " Visit to Surgical Clinics at Home and Abroad," for they will there find an account of the clinics of such well-known surgeons as Sir Wm. Macewen and Professor Tuffier of Paris. Both are written in such a way as to present a very realistic picture of the surgeon at work.

We think the record of " Instructive Mistakes " by surgeons —four of which are given in this number—may prove of much value. They are published anonymously.

There is only one feature of the Journal we venture to think undesirable, and that is the omission of any statement as to the post held by the writer of the article (we do not think the omission of his qualification of any moment). When reading the paper by Mr. R. E. Kelly on " Intratracheal Anæsthesia," we concluded the author was an anæsthetist. We find, however, that he is on the surgical staff of the Liverpool Infirmary. It would have been interesting when reading the article to have known it was written by a surgeon. It seems most fitting that the frontispiece of the Journal should be an excellent picture of Lord Lister, and the first page devoted to a short reference to his life's work.

We very strongly recommend the *British Journal of Surgery* to all British surgeons as one of the best methods of making acquaintance with the advances of surgery, and as a collection of papers of much interest and value.

